# Supplementary material for: Effects of Different Molecular Weight Oxidized Dextran as Crosslinkers on Stability and Antioxidant Capacity of Curcumin-Loaded Nanoparticles
Source: Foods. 2023 Jun 29;12(13):2533. doi: 10.3390/foods12132533 (PMC10341269; doi:10.3390/foods12132533)
Supplement: Supplementary file 1 [file foods-12-02533-s001.zip › foods-2401001-SI.pdf]

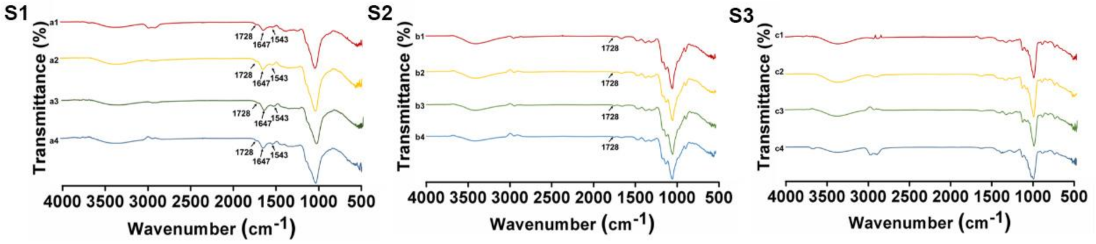

(S1) NPO prepared by other molecular weight (a1: 10 kDa; a2: 40 kDa; a3: 70 kDa; a4: 100 kDa);
(S2) Odex prepared by other molecular weight (b1: 10 kDa; b2: 40 kDa; b3: 70 kDa; b4: 100 kDa);
(S3) dextran with other molecular weight (c1: 10 kDa; c2: 40 kDa; c3: 70 kDa; c4: 100 kDa).

Figure S1. Characterization of NPO, Odex and dextran with different molecular weights.
